# Substantial viral and bacterial diversity at the bat–tick interface

**DOI:** 10.1099/mgen.0.000942

**Published:** 2023-03-02

**Authors:** Ayda Susana Ortiz-Baez, Thomas G. T. Jaenson, Edward C. Holmes, John H.-O. Pettersson, Peter Wilhelmsson

**Affiliations:** ^1^​ Sydney Institute for Infectious Diseases, School of Medical Sciences, the University of Sydney, Sydney, New South Wales 2006, Australia; ^2^​ Department of Organismal Biology, Evolutionary Biology Centre, Uppsala University, SE-752 36, Uppsala, Sweden; ^3^​ Zoonosis Science Center, Department of Medical Biochemistry and Microbiology, University of Uppsala, SE-751 23 Uppsala, Sweden; ^4^​ Clinical Microbiology and Hospital Hygiene, Uppsala University Hospital, 75237 Uppsala, Sweden; ^5^​ Department of Biomedical and Clinical Sciences, Division of Inflammation and Infection, Linköping University, SE-581 83 Linköping, Sweden; ^6^​ Department of Clinical Microbiology, Region Jönköping County, SE-553 05 Jönköping, Sweden

**Keywords:** meta-transcriptomics, *Carios vespertilionis*, *Pipistrellus pygmaeus*, RNA virome, virus evolution

## Abstract

Ticks harbour a high diversity of viruses, bacteria and protozoa. The soft tick *Carios vespertilionis* (Argasidae) is a common ectoparasite of bats in the Palearctic region and is suspected to be vector and reservoir of viruses and other microbial species in bat populations, some of which may act as zoonotic agents for human disease. The Soprano pipistrelle (*Pipistrellus pygmaeus*, Vespertilionidae) is widely distributed in Europe, where it can be found inside or close to human habitation. We used meta-transcriptomic sequencing to determine the RNA virome and common microbiota in blood-fed *C. vespertilionis* ticks collected from a Soprano pipistrelle bat roosting site in south-central Sweden. Our analyses identified 16 viruses from 11 virus families, of which 15 viruses were novel. For the first time in Sweden we identified Issuk-Kul virus, a zoonotic arthropod-borne virus previously associated with outbreaks of acute febrile illness in humans. Probable bat-associated and tick-borne viruses were classified within the families *Nairoviridae, Caliciviridae* and *Hepeviridae*, while other invertebrate-associated viruses included members of the *Dicistroviridae*, *Iflaviridae, Nodaviridae*, *Partitiviridae*, *Permutotetraviridae*, *Polycipiviridae* and *Solemoviridae*. Similarly, we found abundant bacteria in *C. vespertilionis,* including genera with known tick-borne bacteria, such as *Coxiella* spp. and *Rickettsia* spp. These findings demonstrate the remarkable diversity of RNA viruses and bacteria present in *C. vespertilionis* and highlight the importance of bat-associated ectoparasite surveillance as an effective and non-invasive means to track viruses and bacteria circulating in bats and ticks.

## Data Summary

The sequencing reads and viral sequences identified in this study were deposited at the Sequence Read Archive (SRA) database under accession numbers SAMN29627891–SAMN29627902 (Bioproject: PRJNA838788) and GenBank database (OP514647–OP514662; OP804625–OP804628; OP782089–OP782093; OP857220).

**Impact Statement**

Bats and ticks are known vectors and reservoirs of diverse pathogenic and non-pathogenic viruses, bacteria and protozoa. The Soprano pipistrelle is a common bat species in Europe that is often parasitized by the soft tick *Carios vespertilionis*. Given that both the bat and tick can be found in direct proximity to human habitation and are associated with disease-causing zoonotic agents, we used meta-transcriptomic sequencing to uncover the RNA virome and microbiota in ticks that had recently blood-fed off Soprano pipistrelle individuals. In addition to identifying 15 novel viruses and several abundant bacteria, we also detected Issuk-Kul virus, a zoonotic pathogen associated with human disease. Our study not only expands our knowledge of bat–tick-associated viruses and microbes, but also demonstrates the utility and importance of using ectoparasites to non-invasive survey bats for known and novel viruses and bacteria.

## Introduction

The soft tick *Carios vespertilionis,* formerly known as *Argas vespertilionis* (Ixodida: Argasidae) [1], is a common ectoparasite of several bat species in Eurasia and Africa [2, 3]. This soft tick can be found inside or surrounding bat roosts within caves, burrows, wall crevices, tree cavities and other places associated with its hosts. Although *C. vespertilionis* is a bat-specialist [4], it can incidentally feed on birds, domestic dog and humans, and may thus be a vector of zoonotic microorganisms and viruses [5]. During their life cycle, the larvae attach to the infested bat for 14–31 days, while nymphs and adults feed to repletion in about half an hour [6].

Bat species in the family Vespertilionidae*,* the largest within the Chiroptera, are frequent hosts of *C. vespertilionis*. Among these*,* the Soprano pipistrelle (*Pipistrellus pygmaeus*) is an important host species in the Western Palearctic. *P. pygmaeus* is widely distributed in Europe and it is known to congregate in colonies of several hundred. Buildings serve as common sites for roosting while riparian and woodland habitats are preferred for foraging [7, 8]. In Sweden, *P. pygmaeus* occurs in the south and south-central parts of the country, where it is often well adapted to human habitations [5]. In the wild, the diet of pipistrelle bats largely comprises flying Diptera and Lepidoptera. In the IUCN Red List *P. pygmaeus* is classified in the Least Concern category, but roost destruction is a common threat to this bat species [8].

Bats are common reservoirs for zoonotic agents that can potentially be transmitted by their ectoparasites [9]. *C. vespertilionis* has been recorded parasitizing *P. pygmaeus* in Sweden [5, 10] and, although evidence is currently lacking, are suspected to be vectors of bat-associated pathogens, including viruses, bacteria and protozoans. For instance *Borrelia* bacteria, including *Borrelia afzelii *, have been recorded from *C. vespertilionis* [5, 11, 12]. Other tick-borne microorganisms recorded from *C. vespertilionis* include *Rickettsia* spp.*, Ehrlichia* spp. and *Babesia* spp. [9]. However, a lack of virome studies means that only a limited number of viruses have been detected in these ticks to date, including Issyk-Kul virus (ISKV; *Nairoviridae*), Sokuluk virus (SOKV; *Flaviviridae*) and Soft tick bunyavirus [2, 13–15]. Similarly, in the case of *P. pygmaeus* only a few zoonotic viruses within the families *Adenoviridae*, *Astroviridae*, *Coronaviridae* and *Herpesviridae* have been documented [16, 17].

The implementation of bulk RNA-sequencing (meta-transcriptomics) technologies has revolutionized our understanding of the virome diversity and virus–host interactions in nature [18–21]. In particular, the use of meta-transcriptomics has revealed an enormous diversity of RNA viruses in invertebrate species, as well as revealing ancestral evolutionary links to vertebrate RNA viruses [22, 23]. Since there is limited knowledge of the RNA virome of *C. vespertilionis* and *P. pygmaeus*, we investigated what proportion of viruses present in the bat–tick system is either shared between this ectoparasite and its bat host or is specific to each host type. To address this question, we used meta-transcriptomics to determine the virome, as well as common non-viral tick-borne microorganisms, associated with *C. vespertilionis* from a bat-box inhabited by *P. pygmaeus* in south-central Sweden.

## Methods

### Sample collection

Tick specimens of *C. vespertilionis* were collected in the morning from 24 June to 4 August 2020 from a plastic tray placed on the ground below a artificial wooden bat-box housing a colony of about 250–500 adult females and juveniles of *P. pygmaeus* located in a garden at Snesslinge, province of Uppland, South-Central Sweden (60.19.567° N 18.067° E). The nursery bat house was made with eight chambers with dark exterior surfaces to increase attraction to bats [24]. An electric heater was placed in a bat-restricted area of the house for use during very cold nights. Extra holes were included in the walls of the house to allow sufficient air circulation during hot summer days. The bat house was located in an open part of the garden with a mixture of spruce and broadleaf trees. To minimize bats being attacked by predators, the house was placed on poles about 3.5 m above the ground. A total of 165 ticks, naturally detached from the bats, were collected, placed in vials containing RNA later (Thermo Fisher Scientific) and examined microscopically for ingested blood meal. Ticks were identified microscopically to species level and developmental stage (larva, nymph or adult) based on their morphological characters as previously described [25–29]. The ticks were stored in RNA later at −28 °C for 4–6 months and subsequently at −80 °C until molecular analyses.

### Sample preparation and sequencing

Ticks were processed into 12 libraries, pooling between three and 24 individuals of different developmental stages per library (Table S1, available in the online version of this article). Tick samples were homogenized using 0.1 mm ZR BashingBeads (Zymo Research) for 180 s using a bench-top homogenizer (TissueLyzer II; Qiagen). Total RNA was extracted from the homogenates using the ZymoBIOMICS DNA/RNA Miniprep Kit (Zymo Research) according to the manufacturer’s instructions. Library preparation and rRNA depletion were performed using the Tecan Trio RNA-seq kit (NuGEN Technologies), following the manufacturer’s protocol. Bulk paired-end RNA sequencing was performed on the DNBseq platform by the Beijing Genomics Institute (BGI).

### Sequence data processing and assembly

Quality control of sequencing reads was performed with FASTQC [30] and summarized using the MultiQC tool [31]. Reads were *de novo* assembled into contigs using MEGAHIT v1.2.9 with default settings [32]. Assembled contigs were compared against the NCBI non-redundant database (NCBI-nr) using DIAMOND BLASTX with an e-value cut-off ≥1E-4 [33]. To provide an overview of the viral and microbial composition in the ticks, taxonomic profiling was performed using CCMetagen [34]. ORF prediction and protein translation were performed on contigs above 900 nt with the getORF program (EMBOSS). ORFs were predicted as translation regions between STOP codons (-minsize 600 -find 0). Proteins and conserved domains present in the viral contigs were annotated using InterProScan v5.52–86.0 and HMMER v3.3 (*hmmscan* program), with default search parameters [35]. To quantify virus abundance, we filtered out ribosomal reads from Bacteria, Archaea and Eukarya using SortmeRNA v. 2.1b [36], with the non-ribosomal reads then mapped to the virus contigs with BBMap v37.98. Relative contig abundance was computed as the number of reads per million (RPM). To determine the prevalence of the viruses across the samples and avoid false-positives, read counts <0.1 % of the highest abundance for each virus were assumed as the result of index-hopping and removed. Virus abundance was put in context of host gene abundance by comparisons with the mitochondrial 12S and 16S rRNA genes that are stably expressed in *C. vespertilionis* and *P. pygmaeus*. Similarly, we used 16S and 18S rRNA genes to compare sequence abundance in bacteria and protozoa, respectively.

## Microbiota profiling

We focused on targeting the common bacteria and protozoan microbiota found in ticks. To this end, we targeted the 16S rRNA gene for bacterial agents and 18S rRNA for protozoans. When no rRNA genes were detected, unfiltered reads (i.e. prior to rRNA filtering) were mapped against available reference sequences corresponding to *Anaplasma* (NR_044762.1), *Borrelia* (NR_170496.1), *Ehrlichia* (MF069159.1), *Escherichia* (NR_074902.1), *Francisella* (NR_074665.1), *Rickettsia* (NR_074394.1), *Delftia* (NR_116495.1), *Pseudomonas* (NR_117678.1), *Coxiella* (NR_104916.1), *Moraxella* (NR_104936.1) and *Babesia* (AB242176) as these are common tick microbiota components or known mammalian pathogens [3]. The majority consensus sequences were obtained from the most common nucleotides shared between the overlapping reads that mapped to the reference sequences. Consensus sequences were screened against the NCBI nr/nt and rRNA/ITS databases for validation. Further verification of the quality of the rRNA sequences was performed using the Ribovore v1.0.2 software [37]. When no rRNA gene contigs or other suitable marker genes were detected, consensus sequences were only used for phylogenetic contextualization. Abundance was estimated as RPM by mapping reads to the reference sequences as described above.

## Phylogenetic analysis

For each virus taxonomic group, amino acid sequences corresponding to the RNA-dependent RNA polymerase (RdRp) were aligned to reference sequences available in GenBank using the E-INS-I iterative refinement method implemented in MAFFT v7.487 software [38]. Accordingly, the 16S and 18S rRNA marker genes were used for bacteria and protozoans as noted above. The best-fit model of amino acid (coding sequences) and nucleotide (ribosomal sequences) substitution, as well as phylogenetic relationships, were inferred using the maximum-likelihood (ML) method available in IQ-TREE v1.6.12 [39]. Tree node support was estimated with SH-aLRT and the ultrafast bootstrap (UFBoot) [40]. A total of 1000 replicates were run along the ‘bnni’ option to limit branch support overestimation. Tree visualization and annotation was performed using the R packages ggplot2 [41] and Inkscape v1.2 software.

## Virus nomenclature

Novel viruses were provisionally named based on geographical locations within the area (province of Uppland) where the Soprano pipistrelle and the soft tick *C. vespertilionis* are known to occur.

## Results

A total of 165 ticks (144 larvae, 12 nymphs and nine adults) of *C. vespertilionis*, all with visible blood in their guts, were collected from the roost of *P. pygmaeus*. We used a meta-transcriptomics approach to reveal the RNA virome and bacterial components of bat-associated *C. vespertilionis* ticks. In total, we generated ~846 million reads, of which ~101 million corresponded to non-ribosomal reads. Approximately 51 000 contigs were assembled from the total number of reads.

**Fig. 1.**
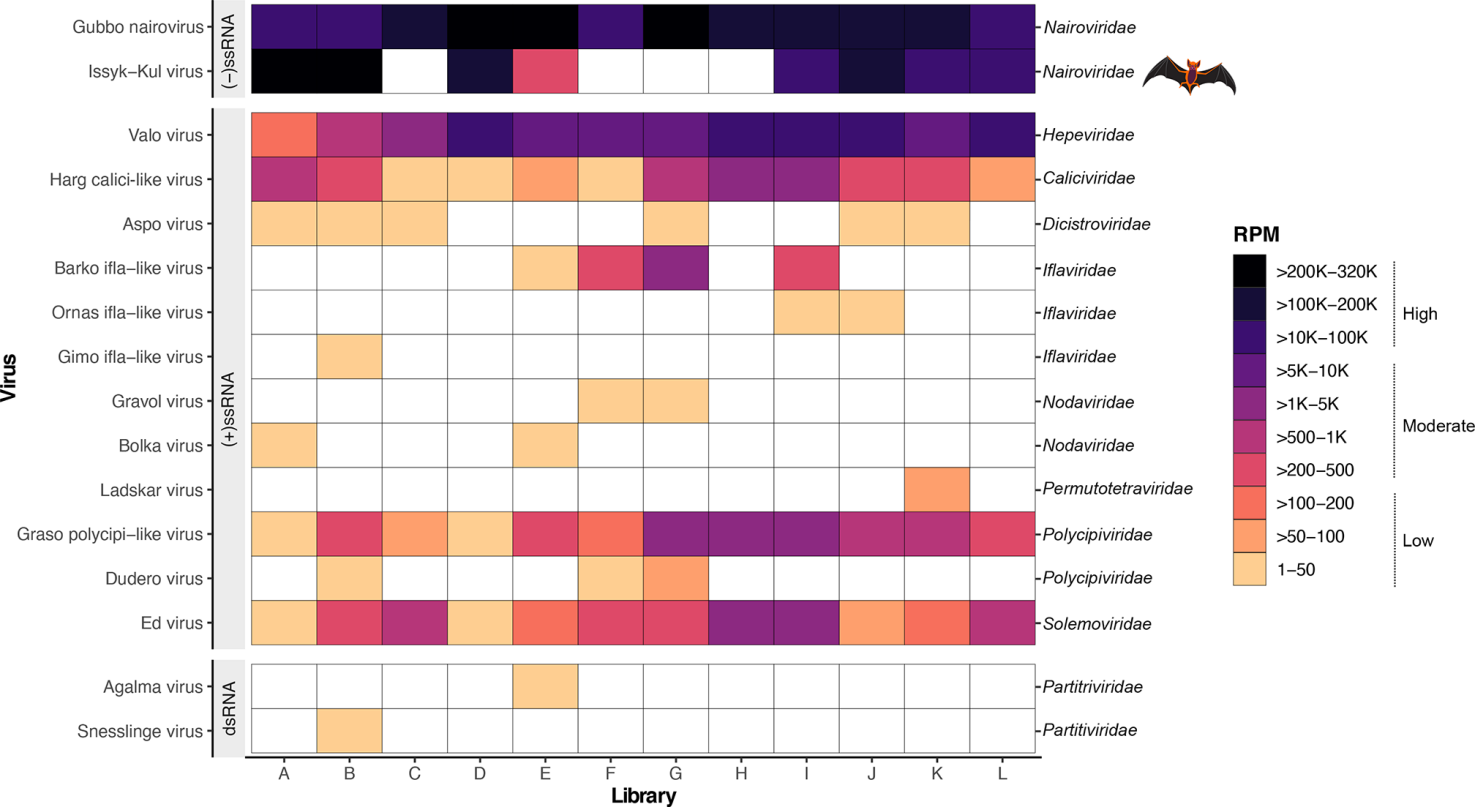
Overview of virus abundance across each bat-associated tick library. Abundance is quantified as the number of reads per million (RPM). RNA viruses are grouped according to the Baltimore classification. Levels of virus abundance are categorized as high, moderate and low, as shown in the key. The bat silhouette indicates whether a virus has previously been identified in bats and only applies to Issyk−Kul virus.

We detected a high diversify of RNA viruses and microbiota, corresponding to bacteria and parasitic protozoa in the bat-ticks analysed. Overall, we identified 16 viruses based on the identification of RdRp sequences, including 15 putative novel viruses within the families *Caliciviridae*, *Dicistroviridae*, *Hepeviridae*, *Iflaviridae, Nairoviridae*, *Nodaviridae*, *Partitiviridae*, *Permutotetraviridae*, *Polycipiviridae* and *Solemoviridae* (Table 1). Among these, we detected at least one bat-associated tick-borne arbovirus within the *Nairoviridae* (Fig. 1). The most abundant families were the *Nairoviridae* and *Hepeviridae*, although the *Polycipiviridae*, *Caliciviridae* and *Solemoviridae* were moderately abundant (Fig. 1). Also of note, we detected three short viral contigs (libraries D and E) that were highly similar to known bat paramyxoviruses (*Paramyxoviridae*), as shown in the blastx similarity search and an associated phylogenetic analysis (Fig. S1, Table S2). Although we excluded all contigs shorter than 900 nt (300 aa) from the analyses, we further characterized these contigs given the probable bat origin and relevance to surveillance. Accordingly, the paramyxovirus-like sequences (381–595 nt) covered different regions in the L protein, including conserved motifs found in the RdRp [SRLF*RNIGDP] and the G-7-mTase [LSHP] domains. Similarly, the contig partially covering the RdRp was assigned with ~36 % similarity and 99.9 % confidence to the RdRp of the parainfluenza virus (Fig. S1, Table S2). The full diversity of RNA viruses characterized in this study included two negative-sense RNA viruses (-ssRNA), 12 positive-sense RNA viruses (+ssRNA) and one double-strand RNA (dsRNA) virus. Likewise, the virus prevalence ranged from six to ten viruses detected per tick library.

**Table 1.** Summary of novel and known RNA viruses identified in this study and their closest hits in the NCBI/nr database

Contig	Provisional name/virus name	Contig length (bp)	Best hit on the NCBI/nr database	Similarity	E-value	Provisional classification	Pools
k99_1661	Harg calici-like virus	9766	UCS96400.1 hypothetical protein 1 [Riboviria sp.]	79.13	0.00E+00	*Caliciviridae*	A, B, C, E, G, H, I, J, K, L
k99_2737	Aspo dicistro-like virus	1290	QPG92983.1 polyprotein [Ohio dicistro-like virus]	65.6	1.28E-185	*Dicistroviridae*	A, B, C, G, J, K
k99_7	Valo virus	4700	QIS88064.1 polyprotein, partial [Bulatov virus]	46.62	0.00E+00	*Hepeviridae*	A, B, C, D, E, F, G, H, I, J, K, L
k99_1852	Barko iflavirus	9948	ACH57393.1 polyprotein [Infectious flacherie virus]	36.5	0.00E+00	*Iflaviridae*	E, F, G, I
k99_2945	Ornas iflavirus	677	QKW94197.1 RNA-dependent RNA polymerase, partial [Sacbrood virus]	54	1.07E-82	*Iflaviridae*	I, J
k99_7939	Gimo ifla-like virus	706	AOY34458.1 polyprotein, partial [Rolda virus]	38.9	5.96E-35	*Iflaviridae*	B
k99_1517	Gubbo nairovirus	12 421	AKC89355.1 RNA-dependent RNA-polymerase, partial [Artashat orthonairovirus]	50.5	0.00E+00	*Nairoviridae*	A, D, E, F, G, H, I, J, K, L
k99_1658	Issyk-Kul virus*	12 288	AKI29982.1 RNA-dependent RNA-polymerase protein [Issyk-Kul virus]	99.7	0.00E+00	*Nairoviridae*	A, B, C, D, E, F, G, H, I, J, K, L
k99_2267	Gravol virus	1074	YP_009337883.1 RNA-dependent RNA polymerase [Hubei orthoptera virus 4]	42.8	1.29E-85	*Nodaviridae*	F, G
k99_1814	Bolka virus	811	NP_077730.1 RNA dependent RNA polymerase protein A [Nodamura virus]	65.9	2.33E-114	*Nodaviridae*	A, E
k99_1453	Agalma virus	751	YP_009342458.1 RdRp [Wuhan fly virus 5]	78.5	6.61E-135	*Partitiviridae*	E
k99_43	Snesslinge virus	1299	BBE15516.1 RNA-dependent RNA polymerase [Osugoroshi virus 1]	73.9	7.22E-200	*Partitiviridae*	B
k99_2789	Ladskar virus	909	AOC55066.1 polyprotein, partial [Niehaus virus]	70.8	1.64E-127	*Permutotetraviridae*	K
k99_543	Graso virus	10 048	QGA87336.1 polyprotein, partial [Hammarskog picorna-like virus]	25.5	1.96E-119	*Polycipiviridae*	A, B, C, E, G, H, I, J, K, L
k99_1507	Dudero virus	919	QHA33683.1 polyprotein [Cacaos virus]	45.2	2.73E-68	*Polycipiviridae*	B, F, G
k99_3888	Ed virus	2639	QEM39297.1 RNA-dependent RNA polymerase [Humaita-Tubiacanga virus]	51	7.36E-149	*Solemoviridae*	A, B, C, E, G, H, I, J, K, L

* Known virus.

### Likely tick-borne and bat-associated virus families

**Fig. 2.**
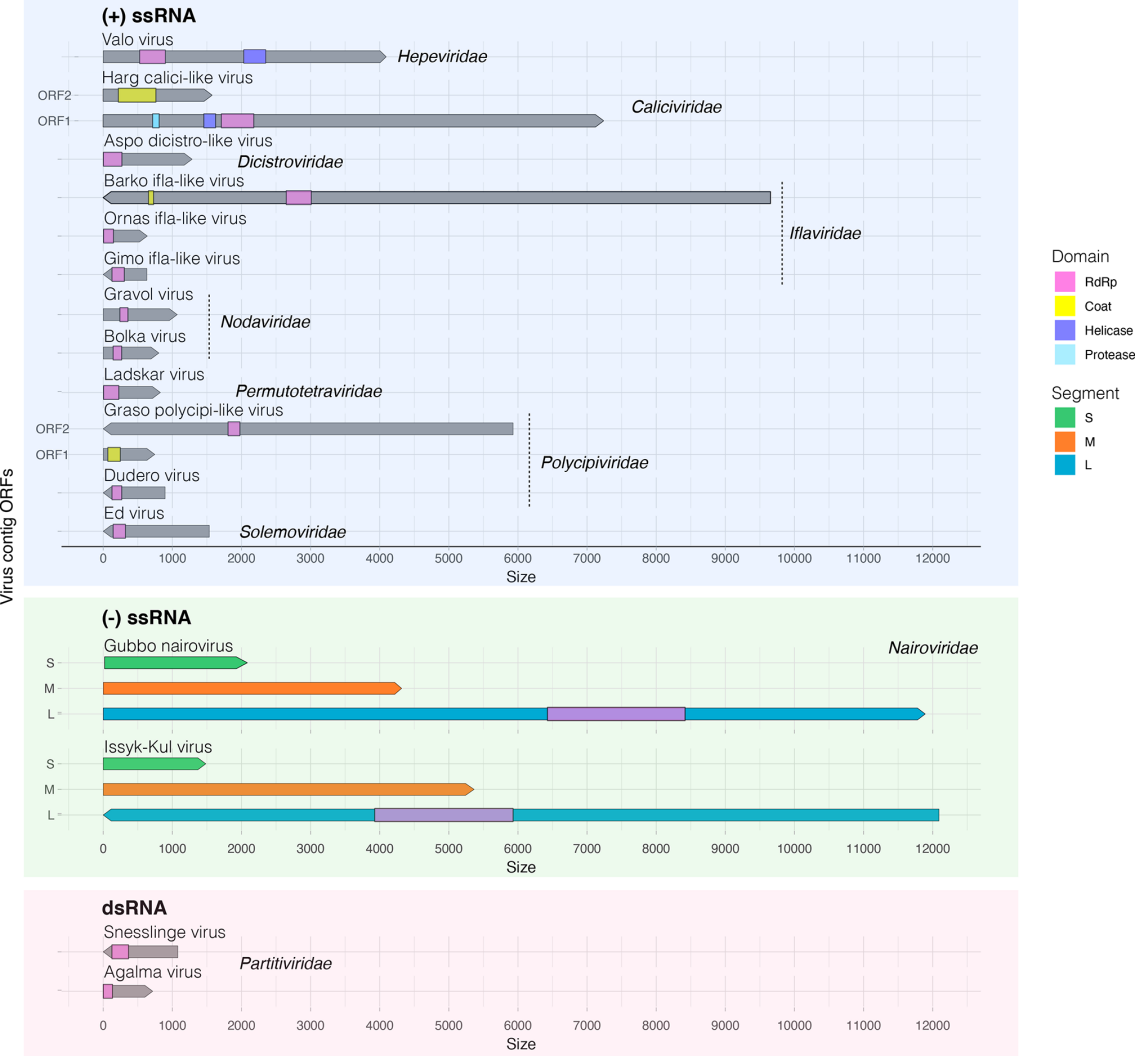
Schematic representation of the ORFs found for the RNA viruses identified in the bat-tick libraries analysed. ORFs are shown as arrow-shaped boxes whose orientation depends on the frames in which they were identified. Domains and segments are indicated as shown in the key.

**Fig. 3.**
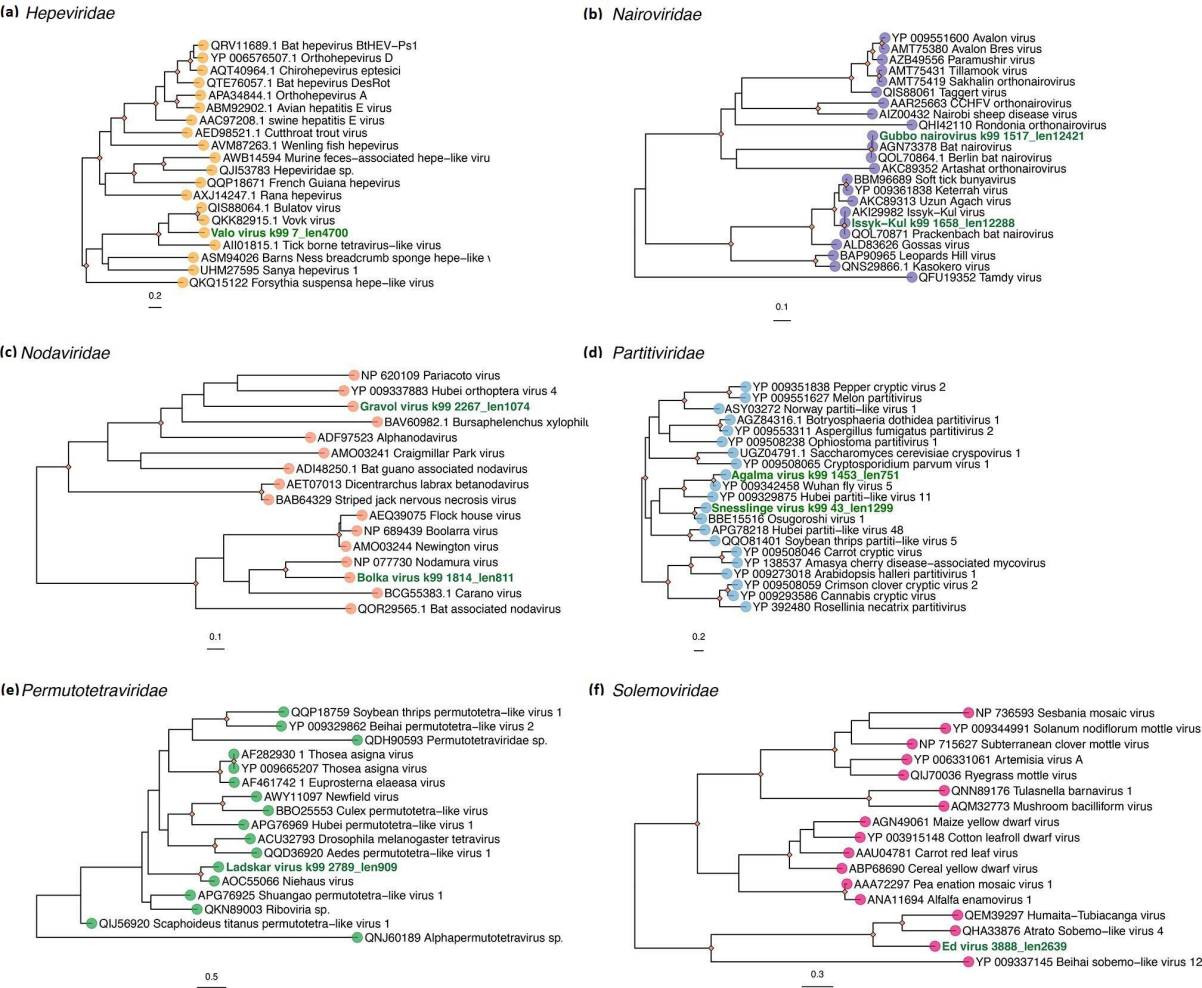
Phylogenetic relationships among the viruses identified in this study and representative background sequences from relevant families of RNA viruses. (**a**) *Hepeviridae,* (**b**) *Nairoviridae*, (**c**) *Nodaviridae,* (**d**) *Partitiviridae*, (**e**) *Permutotetraviridae* and (**f**) *Solemoviridae*. The viruses obtained here are indicated with green. In each case ML trees are mid-point rooted for clarity and were reconstructed based on the amino acid sequences of the RdRp. Nodal support values corresponding to SH-aLRT≥80 % and UFboot≥95 % are displayed with orange diamonds on nodes. The scale bars at the bottom of each tree represent the number of amino acid substitutions per site.

We identified two viruses within the *Nairoviridae*, including one novel virus. The novel virus corresponded to Gubbo nairovirus (GUBV) and exhibited the three segments typical to nairoviruses. GUVB shared a limited amino acid sequence in similarity with Artashat orthonairovirus based on comparison with the viral polymerase (aa %id=50.5) (Table 1). We also detected virus contigs corresponding to the large protein (L segment), glycoprotein (M segment) and nucleoprotein (S segment) of the bat-associated ISKV (RdRp aa %id=99.7) (Fig. 2). Both nairoviruses were detected in >80 % of the samples at high abundance levels (Fig. 1). As expected, these viruses grouped phylogenetically with other known tick-borne and bat-associated viruses (Fig. 3). In particular, GUBV was closely related to bat nairovirus and Berlin bat nairovirus detected in organ tissues from European vespertilionid bats. However, the short available sequences for these viruses (127–147 aa) made it difficult to assign with certainty that these correspond to GUBV.

**Fig. 4.**
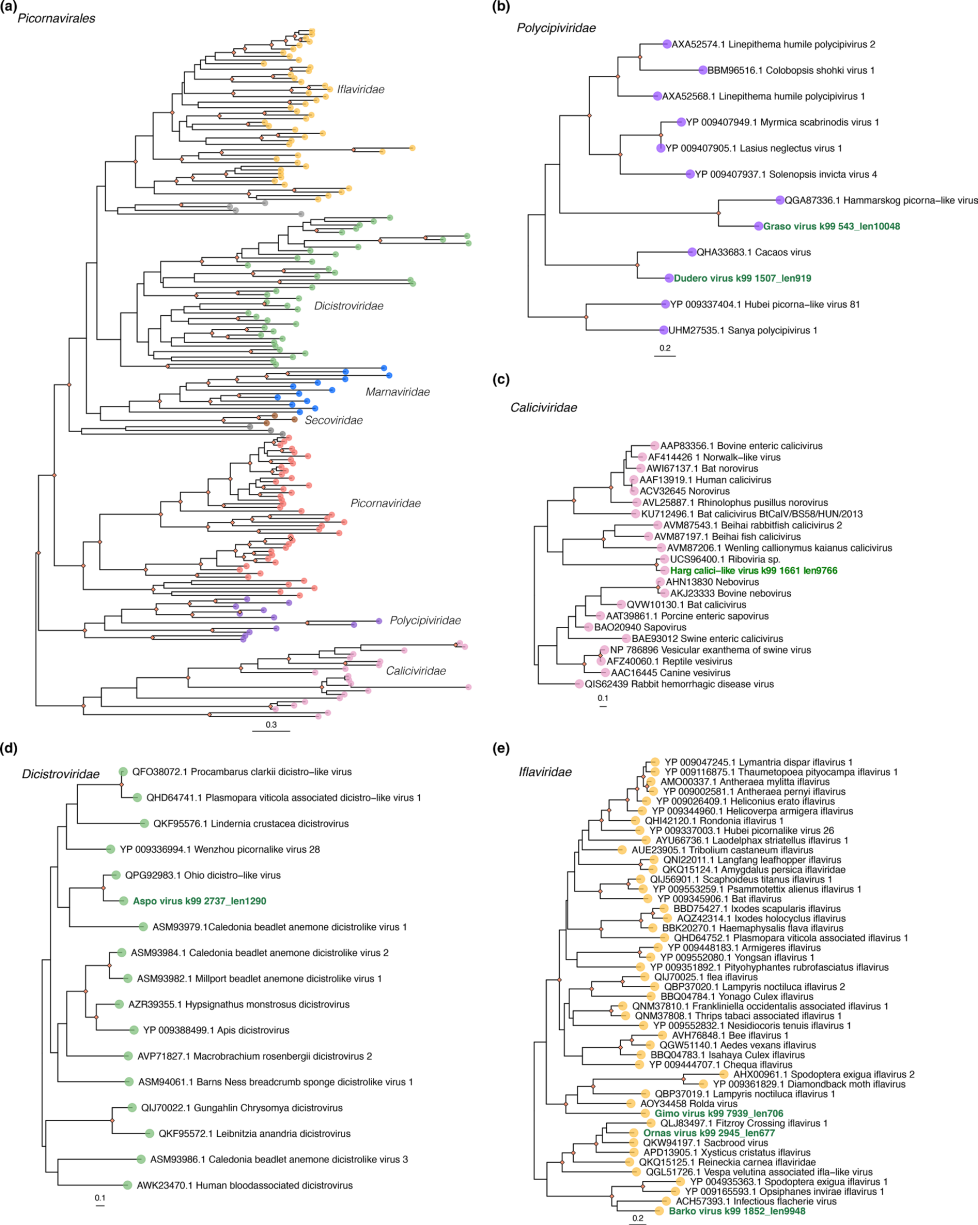
Phylogenetic relationships among the viruses identified in this study and representative background sequences within the (a) *Picornavirales*. The family clades extracted from the order-level tree correspond to the (b) *Polycipiviridae*, (c) *Caliciviridae*, (d) *Discistroviridae* and, (e) *Iflaviridae*. The viruses obtained here are indicated with green-tip labels. In each case ML trees are mid-point rooted for clarity and -reconstructed based on the amino acid sequences of the RdRp. Nodal support values corresponding to SH-aLRT ≥80% and UFboot ≥95% are displayed with orange diamonds on nodes. The scale bars at the bottom of each tree represent the number of amino acid substitutions per site.

Similarly, we identified one novel member from the *Caliciviridae* provisionally referred to as Harg calici-like virus (HCAV), which was present in all the libraries at low to moderate abundance levels (Fig. 1). Phylogenetic analyses showed that HCAV grouped with the unclassified Riboviria sp*.* virus and Clinch calicivirus (Figs 4 and S2), exhibiting above 79 % similarity for the RdRp protein (Table 1, Fig. 4) and 93.3 % similarity for the VP1 protein, respectively. Notably these viruses form a clade basal to taxa of different genera in the *Caliciviridae*. For HCAV we identified the nearly complete genome (~ 9 kb), including two ORFs encoding the RdRp and the major capsid protein VP1, respectively. Finally, among the most abundant viral families, we identified one novel virus – Valo virus (VALV) – belonging to the *Hepeviridae* that was well represented in all the libraries (RPM=200000–100000) (Fig. 1). Phylogenetically, VALV grouped with Bulatov virus and Vovk virus, previously identified in ticks, although it only exhibited 42 % aa sequence similarity to Bulatov virus in the RdRp region as the closest blast hit (Table 1, Figs 2 and 3). As a caveat, abundance levels might be underestimated for partial or shorter virus contigs since RPM estimates are influenced by contig length.

### Likely arthropod and tick microbiome-associated viruses

Among the newly discovered +ssRNA viruses in the *Picornavirales*, we identified three iflaviruses (Barko virus, Ornas virus and Gimo virus), two polycipiviruses (Graso virus and Dudero virus), and one dicistrovirus (Aspo dicistro-like virus). Moreover, we identified two members of the *Nodaviridae* (Gravol virus and Bolka virus), one permutotretavirus (Ladskar virus) and one solemovirus (Ed virus). For all the viruses with the exception of Graso polycipi-like virus and Barko virus, we only detected the viral RdRp gene (Fig. 2). These viruses were most closely related to other arthropod-associated viruses in the different viral families (Table 1, Figs 3 and 4), and were present in low to moderate abundance in the tick libraries analysed. Barko virus, Graso polycipi-like virus and Ed virus were found in higher abundance, while only Graso polycipi-like virus and Ed virus were present in all the libraries (Fig. 1). With respect to the dsRNA viruses, we identified two novel partitiviruses corresponding to Agalma virus and Snesslinge virus based on the presence of a viral RdRp signal (Table 1, Fig. 2). Both viruses were present in a limited number of tick libraries (2/12) at low abundance levels (Fig. 1). The closest relatives were partitiviruses previously found in insects, including Wuhan fly virus 5 and Osugoroshi virus 1 (aa %id=73.9–78.5) (Table 1, Fig. 3).

### Common microbiota in *C. vespertilionis*

**Fig. 5.**
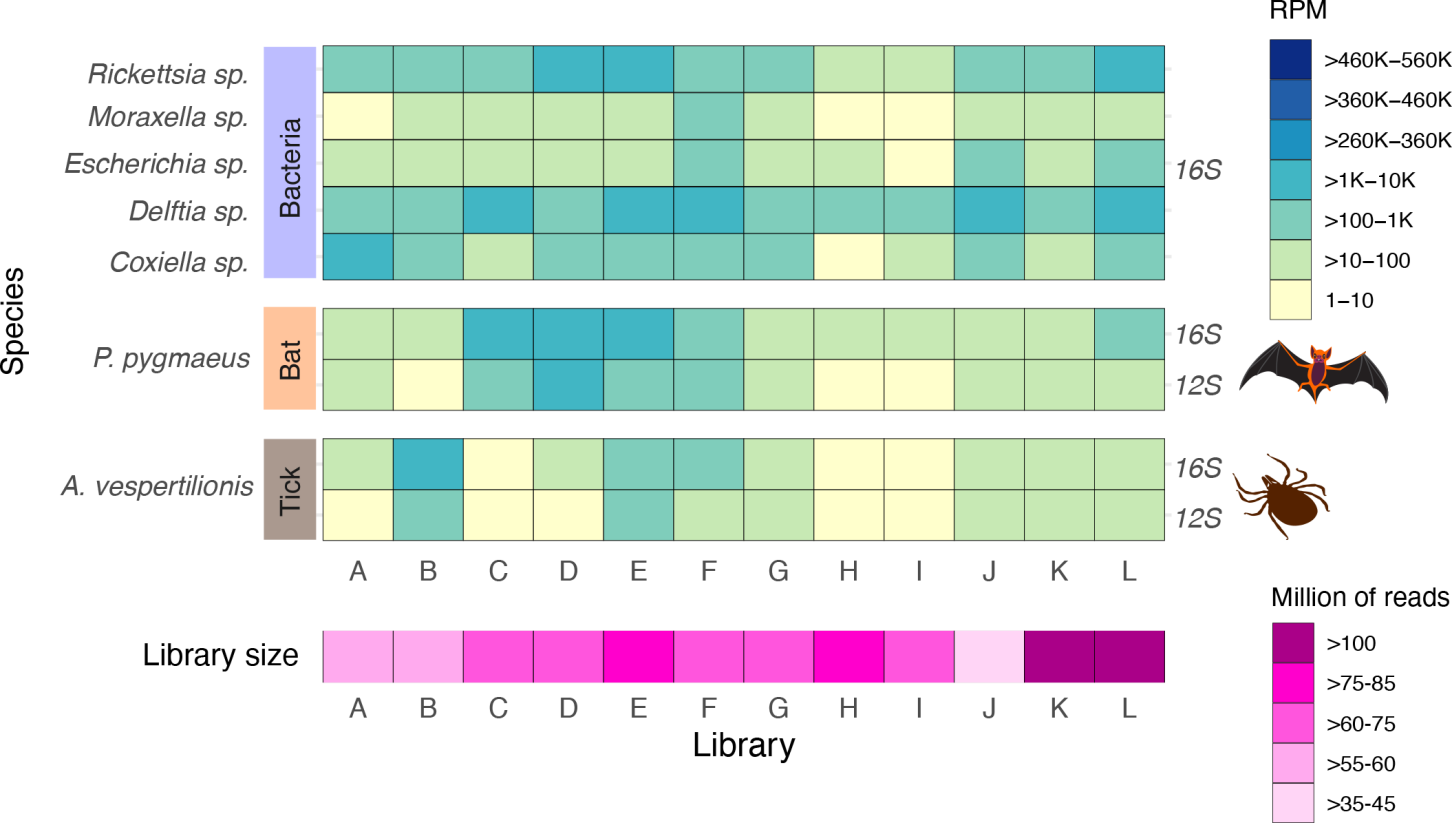
Overview of bacteria abundance across each bat-associated tick library. Abundance is quantified as the number reads per million (RPM) based on the 16S gene. Host expression was assessed using the genes 16S and 12S from *C*. *vespertilionis* and *P. pygmaeus*, as indicated with the animal silhouettes. The bottom panel shows the size across each tick library.

**Fig. 6.**
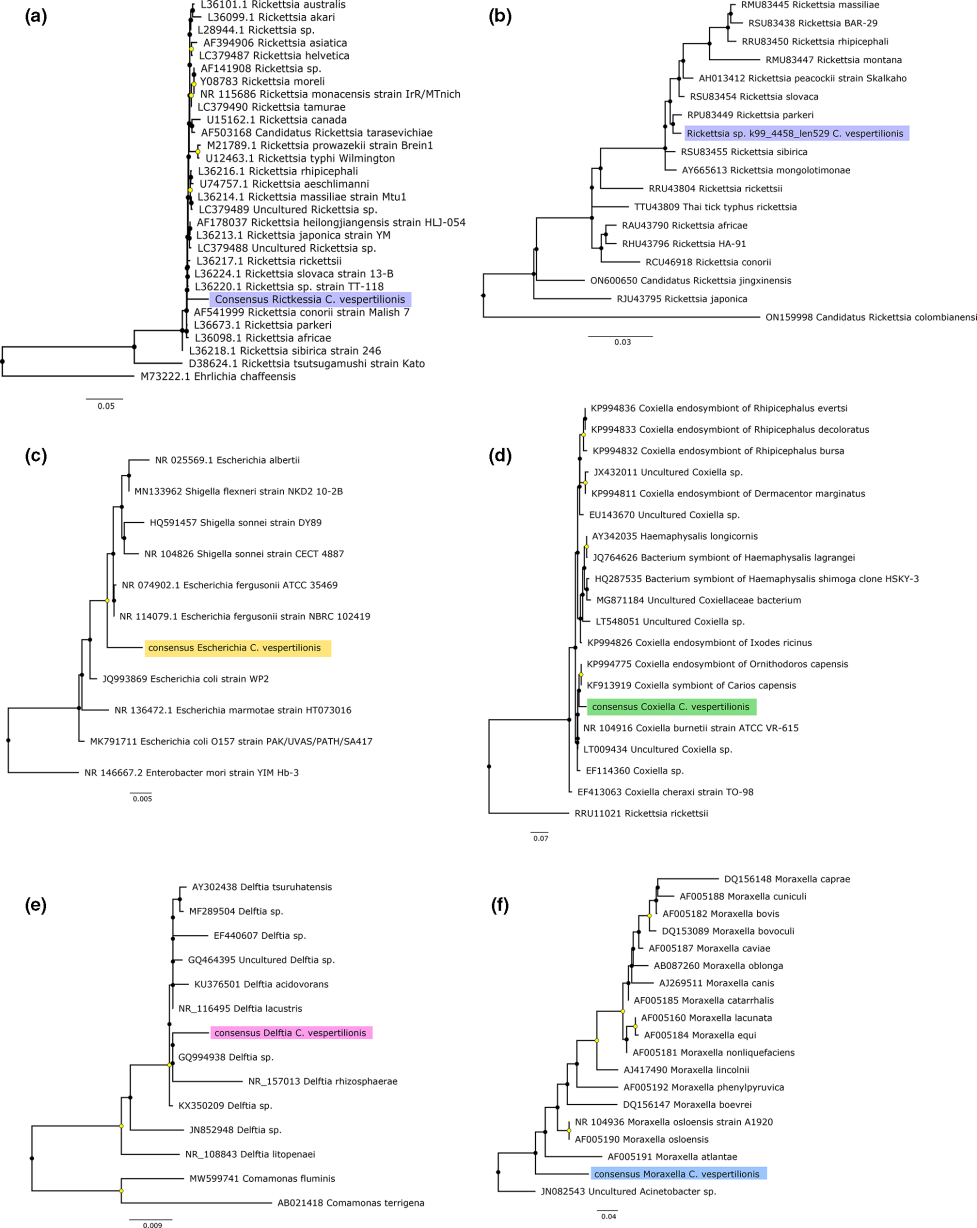
Phylogenetic relationships among the bacterial sequences identified in this study and representative background sequences. The phylogenetic placement of *Rickettsia* was assessed by comparing 16S rRNA (**a**) and *ompA* (**b**) genes, whereas 16S rRNA consensus sequences were used for (**c**) *Escherichia,* (**d**) *Coxiella *, (**e**) *Delftia* and (**f**) *Moraxella *. Bacteria consensus sequences are highlighted in each tree. In each case ML trees are outgroup rooted. Nodal support values corresponding to SH-aLRT≥80 % and UFboot≥95 % are displayed with yellow circles on nodes. The scale bars at the bottom of each tree represent the number of nucleotide substitutions per site.

An analysis of the microbial composition of *C. vespertilionis* revealed the presence of highly abundant tick-borne bacteria genera (~35–66 % of total contigs; taxonomy profiles available at figshare: 10.6084 /m9.figshare.21550899), including members of the *Rickettsia, Delftia* and *Coxiella *, which were present in all the libraries screened (Figs 5 and S3). These bacteria exhibited >97 % similarity at the 16S rRNA gene level to *Rickettsia conorii, Delftia lacustris* and *Coxiella burnetii *, respectively (Table S3, Fig. 6a). In particular, the *Rickettsia* identified here grouped with *Rickettsia* species classified in the spotted fever group (SFG), including *R. conorii *, *R. africae, R. slovaca* and *R. parkeri *. Phylogenetic analysis based on the outer membrane protein A (*ompA*) gene suggested a close relationship to *R. parkeri* within the SFG (~ 99 % nt similarity) (Fig. 6b). In the case of *Coxiella *, we observed close relationships with other microbiota in *Ornithodoros capensis* and *Carios capensis* ticks (Fig. 6). *C. vespertilionis* ticks also harboured other highly prevalent bacteria similar to *Escherichia fergusonii* and *Moraxella osloensis *, although these were detected at much lower abundance levels, and placed as divergent taxa in the phylogenetic tree (Figs 5 and 6, Table S3). We did not detect members of the genera *Anaplasma, Borrelia, Ehrlichia, Francisella* and *Babesia* that were also included in the preliminary screen.

## Discussion

Ticks naturally harbour a highly diverse array of viruses, bacteria and protozoans. Since ticks are obligately haematophagous, these parasitic arthropods might also carry the viruses and microbiota of their hosts acquired during the blood meal [42]. The natural history traits of bat-ticks raise important questions on how the viral and bacterial diversity of ticks is shaped by bat blood meals. In addition, ticks parasitizing bats are of particular interest given that bats are thought to be a natural reservoir for pathogens of veterinary and public health concern [43]. Consequently, ticks might also act as vectors of pathogens circulating in bats, posing a risk for the health of animal populations, including humans. Thus, investigating the diversity of RNA viruses and bacteria in bat-associated ticks could provide a strategy for regular active surveillance of bat-borne zoonoses.

Our analysis of the bat-tick *C. vespertilionis* virome revealed the family *Nairoviridae* (*Bunyavirales*) as the most abundant and prevalent in the libraries of recently blood-fed ticks (Fig. 1). Similar findings have been reported in recent metagenomic studies on different tick and host species across a variety of geographical locations [44–47], suggesting that ticks might be competent hosts and vectors for the replication and transmission of nairoviruses in nature. Among the members of the *Nairoviridae* found here, we identified ISKV [48, 49], a zoonotic virus associated with outbreaks of acute febrile illness in humans. ISKV was originally discovered in Central Asia in the 1970s, but has recently also been recorded in Germany [49–51]. The virus was first identified in a *Nyctalus noctula* bat, although its host range has been expanded to other bat species [49–53]. Similarly, there are reports of ISKV detected in *C. vespertilionis* [49, 52]. Herein, we demonstrate for the first time the presence of ISKV at high abundance levels in bat-ticks in Sweden (Fig. 1). From our current knowledge, there are no reports of ISKV in *P. pygmaeus* bats. However, the presence of ISKV in recently blood-fed *C. vespertilionis* bat-ticks, as well as in other vespertilionid bats, make it plausible that this virus also occurs in *P. pygmaeus*.

The recent detection of the novel GUBV at similar abundance levels to ISKV is compatible with the notion that it might be both a tick-borne and bat-associated virus (Fig. 1). This is also supported by the close relationship of GUBV to other nairoviruses isolated from European bats, suggesting that it might also be able to infect *P. pygmaeus* bats. Since GUBV is distantly related to Artashat orthonairovirus, it might represent a new species within the *Nairoviridae* together with bat nairovirus and Berlin bat nairovirus (Table 1, Fig. 3). Notably, our analysis of the abundance, prevalence and host range of GUBV is limited to a small number of tick samples (Fig. 1, Table S1). Similarly, we cannot exclude the possibility of high viral loads in viraemic bat hosts. Comparative research targeting unfed questing tick and bat samples separately could help test these hypotheses more rigorously. The zoonotic potential and public health significance of GUBV for animal populations similarly merits additional investigation. In combination with previous research [48, 50, 54], our results support the hypothesis that these ticks might serve as vectors and/or potential reservoirs for these nairoviruses.

Although we did identify paramyxovirus-related sequences in the data generated here (Fig. S1, Table S2), they were not included in our analyses due to the limited length of the contigs. It should be noted, however, that paramyxoviruses have been reported in *Pipistrellus* species [51, 55–58]. That we only recovered a few short paramyxo-like sequences from bat-associated ticks might indicate low viral loads in the blood meal [59], and bat urine and faeces may be more suitable samples for the detection of these viruses [55–57].

The presence of newly discovered +ssRNA virus members within the *Caliciviridae* and *Hepeviridae* is consistent with previous research on bat-borne and tick-borne viruses. A few bat caliciviruses (sapoviruses and unclassified viruses) have been discovered in European vespertilionid bats [60, 61], although there are no corresponding reports of viruses in *P. pygmaeus* [16, 17]. The calicivirus identified in this study (HCAV) was highly divergent and unrelated to other bat caliciviruses, with its closest relative being an unclassified virus found in reptile faeces (RdRp, MZ375209) (Fig. 4). Notably, there is no current evidence of ticks carrying caliciviruses or playing a role on their transmission. Based on the divergent phylogenetic position of HCAV, it might represent a member of a new genus within the family *Caliciviridae*, although establishing a definitive association with vertebrate/invertebrate hosts is uncertain. In contrast, the novel hepevirus VALV suspected to be associated with the tick virome was most closely related to Bulatov virus and Vovk virus that have been associated with the virome of *Ixodes uriae* ticks from the Antarctic peninsula [46, 62]. In addition, the high prevalence and abundance of VALV in all the libraries tentatively suggests that the virus replicates in ticks (Fig. 1). Finally, although some hepeviruses have been detected circulating in bats [63–65], our study lacks data to assess if VALV has any association with transmission or disease in bats.

As expected, a considerable fraction of the tick virome corresponded to viruses associated with invertebrates. This included virus families such as the *Iflaviridae, Nodaviridae, Solemoviridae* and *Partitiviridae*, previously identified in the virome of different tick species [44, 66–69]. We also found members of the *Dicistroviridae*, *Permutotetraviridae* and *Polycipiviridae* that are probably infecting the bat-ticks. Indeed, the dominance of Graso polycipi-like virus (*Polycipiviridae*) and Ed virus (*Solemoviridae*) in the tick libraries might indicate the efficient replication of these viruses within this arthropod species, although this will need further research. Similarly, the occurrence of *Partitiviridae* in *C. vespertilionis* is compatible with studies suggesting that partitiviruses can possibly infect arthropods [44, 67, 70, 71], as well as fungi and protozoa in these ectoparasites. However, we were not able to definitively determine the host of these viruses. Previous research on *C. vespertilionis* has been largely focused on targeting tick-borne viruses of public health relevance [52, 72], with the RNA virome as a whole largely unexplored. As such, our work provides a baseline for the study of RNA viruses in *C. vespertilionis*.

It has previously been shown that *C. vespertilionis* can harbour a repertoire of bacterial and protozoal species [73–75]. We identified sequences related to the most common microbial agents in bat-ticks, some of which are of particular interest due to their high abundance (Figs 5 and 6, Table S3). For instance, our data revealed the presence of *Rickettsia* spp. in *C. vespertilionis* collected in Sweden, corroborating previous reports in Europe [9, 73]. Although we were unable to provide a species-level classification based on the 16S rRNA and *ompA* genes, the close relationship to *Rickettsia* species, and in particular to *R. parkeri *, in the SFG might constitute a risk for vector-borne zoonotic disease. Rickettsial infections with some species within the SFG have been associated with pathogenicity in humans [9, 76, 77]. For instance, *R. parkeri* is an emergent tick-borne pathogen and the causative agent of *R. parkeri* rickettsiosis in America [78, 79]. Similarly, in Sweden, infections with *R. helvetica* and *R. felis* have been associated with severe clinical manifestations, including meningitis [77, 80]. In contrast, it has been shown that *Rickettsia* could play a role in the provision of folate in *Ixodes pacificus* ticks [81]. The range of interactions between *Rickettsia* and *C. vespertilionis* remains uncertain, as does their pathogenic potential for bat hosts [73]. Likewise, *Rickettsia* spp. have been reported in bat tissue samples collected from vespertilionid bats [82, 83]. Therefore, whether bacterial infection can impact bat health or whether bats contribute to the maintenance of *Rickettsia* spp. in nature merits investigation [83].

We also reported the co-occurrence of bacteria such as *Delftia* spp. and *Coxiella* spp. (Figs 5 and 6). *Delftia* spp. have been reported as core bacteria in the microbiome of *Dermacentor variabilis* [84]. Given the high abundance and prevalence of *Delftia* spp. in the tick libraries, a similar situation might exist for *C. vespertilionis* [74]. An earlier study documented the presence of *Coxiella burnetii *, the aetiological agent of Q fever, in *C. vespertilionis* ticks collected from Asia [75], although many *Coxiella* species are considered obligate and associated with nutritional and reproductive roles in ticks [85–89]. General questions that remain are whether ticks act as vectors or reservoirs (or both) of all these agents, and what extent the blood meal and the environment contribute to the viral and bacterial composition in bat-ticks.

Overall, we provide new insights into the viral and bacterial diversity associated with *C. vespertilionis* ticks in Sweden. The presence of dominant and underrepresented viruses and bacteria warrants further research into the nature of bat–tick interactions and how these impact viral and microbial transmission. Additional vector competence studies are required to demonstrate that *C. vespertilionis* ticks can become infected when feeding on an infectious host and maintain the pathogen such that it is capable of being transmitted to an uninfected, susceptible host [90]. Despite the small sample size, our study demonstrates that bat-tick surveillance provides an effective and non-invasive means to detect bat- and tick-borne microorganisms circulating in bat roosting habitats. These results reinforce the notion of protecting the natural environment of bats and minimizing human exposure to bat/tick habitats to prevent zoonotic spillover events [91, 92].

## Supplementary Data

Supplementary material 1

Supplementary material 2

Supplementary material 3

Supplementary material 4

Supplementary material 5

Supplementary material 6
