# Supplementary figures and images for: Substantial viral and bacterial diversity at the bat–tick interface
Source: Microb Genom. 2023 Mar 2;9(3):mgen000942. doi: 10.1099/mgen.0.000942 (PMC10132063; doi:10.1099/mgen.0.000942)

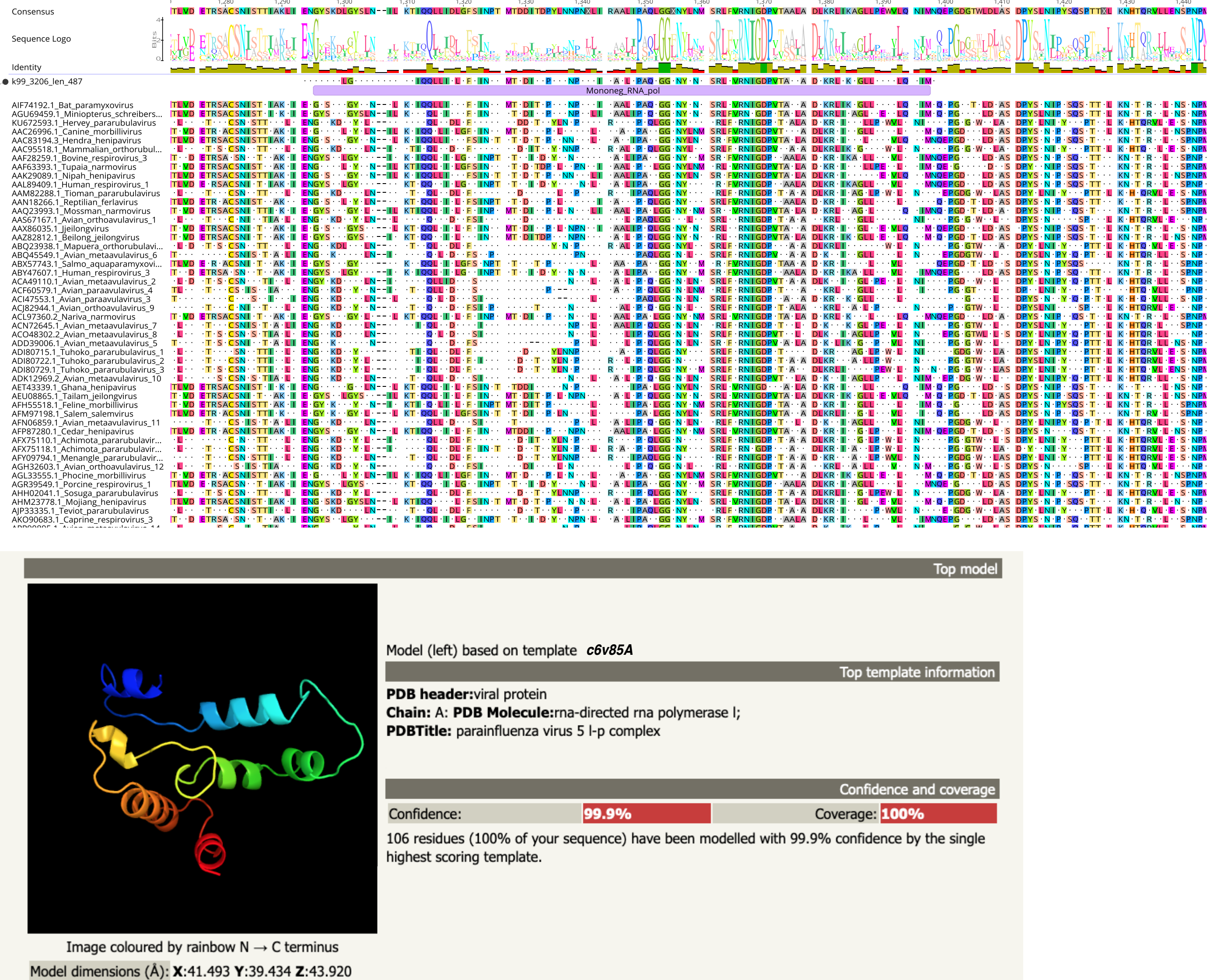

s are shown in the  
are displayed with

Supplement: Supplementary material 1 [file mgen-9-942-s001.pdf]
