# Supplementary material for: Substantial viral and bacterial diversity at the bat–tick interface
Source: Microb Genom. 2023 Mar 2;9(3):mgen000942. doi: 10.1099/mgen.0.000942 (PMC10132063; doi:10.1099/mgen.0.000942)
Supplement: Supplementary material 2 [file mgen-9-942-s002.pdf]

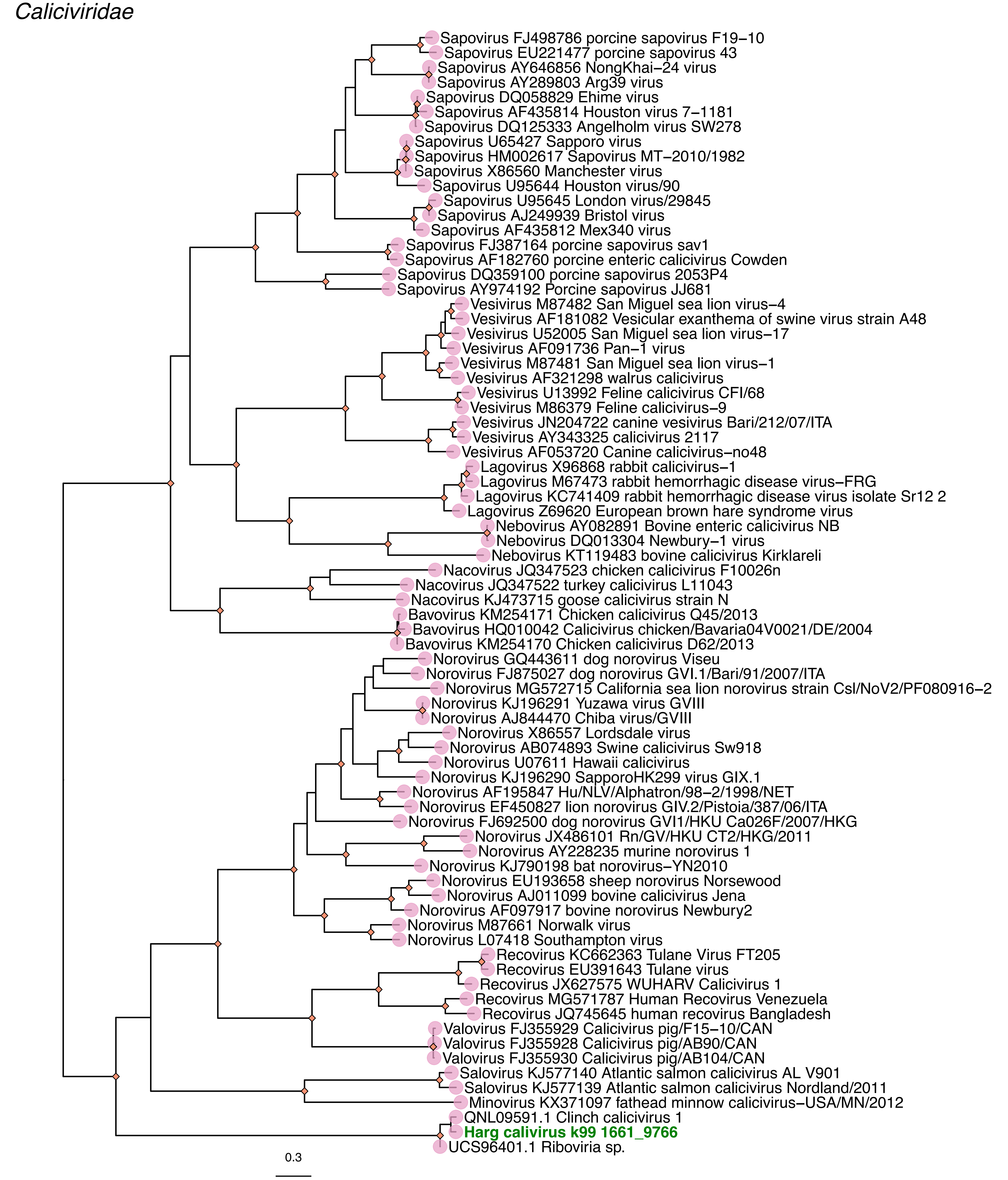

Figure S2. Phylogenetic relationships among the calici-like virus identified in this study and representative sequences within the *Caliciviridae*. The novel virus obtained here is shown in bold green. The maximum-likelihood tree is mid-point rooted for clarity and was constructed based on reference sequences of the VP1 protein (source: https://talk.ictvonline.org). Nodal support values corresponding to SH-aLRT >= 80% and UFboot >= 95% are displayed with orange diamonds on nodes. The scale bars are shown at the bottom of each tree and represent the number of amino acid substitutions per site.
