# Supplementary material for: Substantial viral and bacterial diversity at the bat–tick interface
Source: Microb Genom. 2023 Mar 2;9(3):mgen000942. doi: 10.1099/mgen.0.000942 (PMC10132063; doi:10.1099/mgen.0.000942)
Supplement: Supplementary material 3 [file mgen-9-942-s003.pdf]

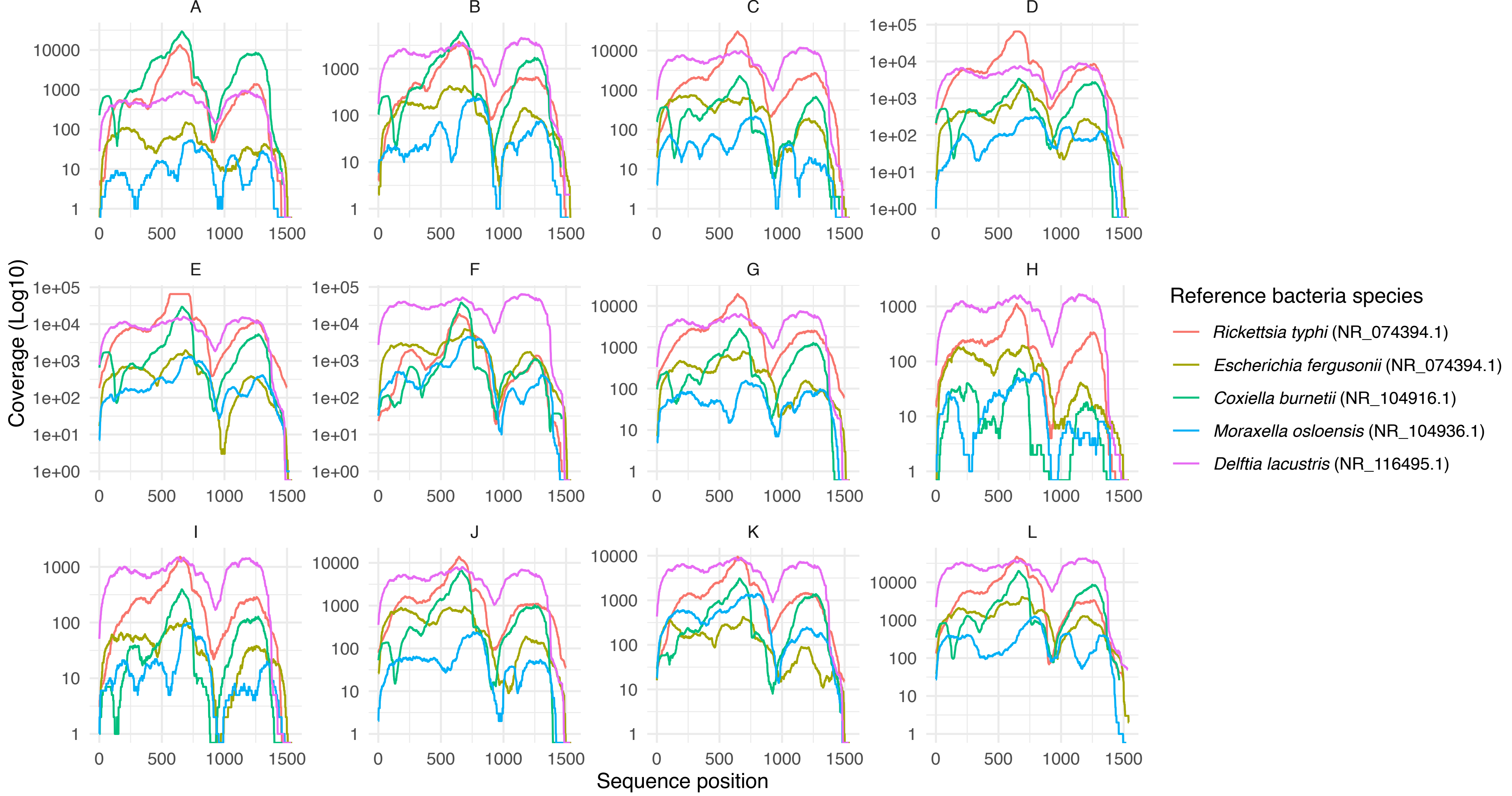

Figure S3. Map coverage plots for the 16S rRNA gene of the bacteria detected across libraries of *Carios vespertilionis* (panels A–L). The reference sequences used here are represented with colors and the GenBank codes are indicated between parenthesis as shown in the key.
