# Supplementary material for: Substantial viral and bacterial diversity at the bat–tick interface
Source: Microb Genom. 2023 Mar 2;9(3):mgen000942. doi: 10.1099/mgen.0.000942 (PMC10132063; doi:10.1099/mgen.0.000942)
Supplement: Supplementary material 4 [file mgen-9-942-s004.pdf]

Table S1. Number of individuals and developmental stages of bat-ticks included in this study.

Pools	# Individuals	Stage
A	3	Adults
B	3	Adults
C	3	Adults
D	4	Nymphs
E	4	Nymphs
F	4	Nymphs
G	24	Larvae
H	24	Larvae
I	24	Larvae
J	24	Larvae
K	24	Larvae
L	24	Larvae
