# Supplementary material for: Substantial viral and bacterial diversity at the bat–tick interface
Source: Microb Genom. 2023 Mar 2;9(3):mgen000942. doi: 10.1099/mgen.0.000942 (PMC10132063; doi:10.1099/mgen.0.000942)
Supplement: Supplementary material 5 [file mgen-9-942-s005.pdf]

Table S2. Detection of paramyxo-like viral sequences and their closest hits in the NCBI/nr database.

Contig	Contig length	Best hit on the NCBI/nr database	Similarity	E-value	Provisional classification	Pool
k99_5075	381	AIF74192.1 polymerase, partial [Bat paramyxovirus]	78.4	1.34E-63	*Paramyxoviridae*	D
k99_3206	487	AIF74192.1 polymerase, partial [Bat paramyxovirus]	90.8	3.95e-44	*Paramyxoviridae*	D
k99_8678	595	AGU69459.1 large protein, partial [Miniopterus schreibersii paramyxovirus]	51.5	1.39E-54	*Paramyxoviridae*	E
