# Supplementary material for: Substantial viral and bacterial diversity at the bat–tick interface
Source: Microb Genom. 2023 Mar 2;9(3):mgen000942. doi: 10.1099/mgen.0.000942 (PMC10132063; doi:10.1099/mgen.0.000942)
Supplement: Supplementary material 6 [file mgen-9-942-s006.pdf]

Table S3. Overview of the closest BLAST hits in the NCBI/16S rRNA database for the bacterial consensus sequences generated in this study.

Best hit on the NCBI/nr database	Sequence length	Similarity	E-value
Rickettsia conorii strain Malish 7 16S ribosomal RNA, partial sequence	1498	97.39%	0.00E+00
Coxiella burnetii strain ATCC VR-615 16S ribosomal RNA, partial sequence	1465	97.14%	0.00E+00
Delftia lacustris strain 332 16S ribosomal RNA, partial sequence	1534	99.13%	0.00E+00
Moraxella osloensis strain A1920 16S ribosomal RNA, partial sequence	1522	92.78%	0.00E+00
Escherichia fergusonii ATCC 35469 16S ribosomal RNA, complete sequence	1542	99.47%	0.00E+00
